# Waist-to-height ratio and cardiometabolic risk factors in adolescence: findings from a prospective birth cohort

**DOI:** 10.1111/j.2047-6310.2013.00192.x

**Published:** 2013-07-25

**Authors:** L Graves, S P Garnett, C T Cowell, L A Baur, A Ness, N Sattar, D A Lawlor

**Affiliations:** 1 Sydney Medical School, University of SydneySydney, Australia; 2 The Children’s Hospital at Westmead Clinical School, University of SydneySydney, Australia; 3 Kids Research Institute, The Children’s Hospital at WestmeadWestmead, Australia; 4 Institute of Endocrinology and Diabetes, The Children’s Hospital at WestmeadWestmead, Australia; 5 School of Oral and Dental Sciences, University of BristolBristol, UK; 6 Metabolic Medicine, University of GlasgowGlasgow, UK; 7 MRC Centre for Causal Analyses in Translational Epidemiology, School of Social and Community Medicine, University of BristolBristol, UK

**Keywords:** Waist-to-height ratio, cardiometabolic risk, ALSPAC longitudinal study

## Abstract

**Objective:**

To examine the associations between body mass index (BMI) and waist-to-height ratio (WHtR) measured in childhood and adolescence and cardiometabolic risk factors in adolescence.

**Methods:**

Secondary data analysis of the Avon Longitudinal Study of Parents and Children, a population based cohort. Data from 2858 adolescents aged 15.5 (standard deviation 0.4) years and 2710 of these participants as children aged 7–9 years were used in this analysis. Outcome measures were cardiometabolic risk factors, including triglycerides, low density lipoprotein cholesterol, high density lipoprotein cholesterol, insulin, glucose and blood pressure at 15 years of age.

**Results:**

Both BMI and WHtR measured at ages 7–9 years and at age 15 years were associated with cardiometabolic risk factors in adolescents. A WHtR ≥0.5 at 7–9 years increased the odds by 4.6 [95% confidence interval 2.6 to 8.1] for males and 1.6 [0.7 to 3.9] for females of having three or more cardiometabolic risk factors in adolescence. Cross-sectional analysis indicated that adolescents who had a WHtR ≥0.5, the odds ratio of having three or more cardiometabolic risk factors was 6.8 [4.4 to 10.6] for males and 3.8 [2.3 to 6.3] for females. The WHtR cut-point was highly specific in identifying cardiometabolic risk co-occurrence in male children and adolescents as well as female children (90 to 95%), but had poor sensitivity (17 to 53%). Similar associations were observed when BMI was used to define excess adiposity.

**Conclusions:**

WHtR is a simple alternative to age and sex adjusted BMI for assessing cardiometabolic risk in adolescents.

**What is already known about this subject**

- In adults, associations between body mass index (BMI), waist-to-height ratio (WHtR) and cardiometabolic outcomes are similar.
- In children and adolescents, results from cross-sectional studies examining the associations between BMI z scores, WHtR and cardiometabolic outcomes are conflicting and there is a paucity of prospective data.

**What this study adds**

- This is the first study to demonstrate the *prospective* association between WHtR in childhood and cardiometabolic outcomes in adolescent boys.
- WHtR is a simple calculation that can be used to identify children and adolescents for cardiometabolic risk without the need for reference growth charts.
- The WHtR cut-point of ≥0.5 was highly specific in identifying cardiometabolic risk co-occurrence but has poor sensitivity.

## Introduction

Obesity in childhood is associated with adverse levels of cardiometabolic risk factors, including higher blood pressure (BP), triglycerides, total and low density lipoprotein cholesterol (LDLc) and insulin, and lower high density lipoprotein cholesterol (HDLc) 1–3. Furthermore, childhood obesity is positively and linearly associated with obesity and related cardiovascular disease in adulthood 4. It is important to identify children who are at increased risk of developing comorbidities associated with obesity, to potentially intervene and prevent the development of chronic disease including type 2 diabetes.

We have previously reported, using data from the Avon Longitudinal Study of Parents and Children (ALSPAC), that childhood body mass index (BMI), waist circumference and total fat mass are positively associated with cardiovascular risk factors in adolescence and the magnitudes of these associations are similar for all measures of adiposity 2. There has been recent interest in the use of the waist-to-height ratio (WHtR) for identifying excessive central adiposity in children and adolescents 5–8. It has been suggested that a WHtR ≥0.5, irrespective of age, sex or ethnicity 5,7,9, is a valid predictor of higher cardiometabolic risk 10–12. WHtR may be a more straightforward anthropometric index to apply in the clinical setting where BMI centile charts may not be readily available.

There have been two systematic reviews in adults examining the associations between BMI and WHtR and cardiometabolic outcomes which have indicated broadly similar associations between the different anthropometric measures and cardiometabolic risk factors 7,13. However, the utility of WHtR in identifying young people at cardiometabolic risk is unclear. Results from cross-sectional studies are conflicting some indicate that WHtR is more strongly associated with cardiometabolic risk factors than BMI 10,12,14–16, while others have found either a weaker 17 or a similar 18,19 association. To our knowledge, there is only one prospective study looking at this issue in children and adolescents. That study reported similar correlation between BMI and WHtR assessed in children when they were aged 7 to 15 years and the metabolic syndrome in young adulthood 20.

Therefore the aims of this study were to:

1. Examine the cross-sectional association between WHtR and cardiometabolic risk factors in adolescents.
2. Examine the prospective association between WHtR assessed in childhood and cardiometabolic risk factors assessed in adolescence.
3. Examine these associations in relevance to the respective associations with BMI.

## Methods

### Participants

This study is a secondary analysis of data from the ALSPAC (http://www.bristol.ac.uk/alspac). The design and methods have been published 21. In brief, ALSPAC is a longitudinal population-based cohort that recruited 14 541 pregnant women who were expected to deliver between 1 April 1991 and 31 December 1992 21. All members of the ALSPAC cohort were invited to annual follow-up clinics from the age of 7 to 13 years and then approximately every 2 years thereafter. In addition, when the oldest children were approximately 7 years of age, the sample size was increased by inviting eligible cases who had failed to join the study originally resulting in an additional 713 children being enrolled; 8297, 7725 and 5509 participants attended the 7-, 9- and 15-year clinics, respectively. In the present study, baseline ‘childhood’ anthropometric data were taken from a combination of the 7- and 9-year clinics. If available, data from the 7-year clinic were used preferentially (*n* = 2540) and data from an additional 168 children were used from the 9-year clinic. Anthropometric and cardiometabolic risk factors were taken from the 15-year clinic (*n* = 2858): the ‘adolescent’ group. The eligibility criteria for these analyses were adolescents who had their waist circumference, height and serum lipid levels measured at the 15 year clinic. There were no childhood anthropometric measurements for 150 adolescents who had both anthropometric and cardiometabolic risk factors measured at the 15-year clinic; consequently, data are missing for 5% (148 of 2858) of the children. The final sample used in the analyses here were 2858 adolescents (cross-sectional analysis) and 2710 children (prospective analysis). The majority (98%) of the mothers of the participants self-identified their ethnicity as white. Ethical approval for the study was obtained from the ALSPAC Ethics and Law Committee and the Local Research Ethics Committees.

### Anthropometry

Standard protocols for assessing anthropometry were used, with the participants in light clothing and no shoes. Age was recorded in months. Weight was measured to the nearest 0.1 kg using Tanita THF 300GS (Tanita UK Ltd, Yewsley, Middlesex, UK). Height was measured using a Harpenden stadiometer (Holtain Ltd, Crymych, Pembs, UK) to the nearest 1 mm. Waist circumference was measured at the mid-point between the lower rib and the iliac crest to the nearest 1 mm with a flexible tape measure. Height, weight and BMI z scores were determined using age and sex specific national reference values for the UK 22. WHtR was determined by dividing waist circumference by height.

### Cardiometabolic risk factors

The participants were requested to fast before attending the 15-year clinic. For those attending morning clinics they were asked to fast overnight. For those attending afternoon clinics, they were asked to fast for a minimum of 6 h prior to attendance. Blood samples were taken from the cubital fossa and immediately spun and plasma was frozen at −80°C. Approximately 3 to 9 months later, the samples were assayed. Total cholesterol, triglyceride and HDLc concentrations were measured using a modified Lipid Research Clinics Protocol with enzymatic reagents for lipid determination 2. LDLc was determined with the Friedewald equation 23. An automated assay was used to measure blood glucose concentration. Insulin was measured using an enzyme-linked immunosorbent assay (Mercodia, Uppsala, Sweden) which does not cross react with proinsulin. BP was measured using a Dinamap 9301 Vital Signs Monitor (Morton Medical, London, UK) with appropriate cuff size. Each participant was at rest and his/her arm was supported. For analysis, the mean of the two measurements that were taken was used.

### Statistical analysis

Data were analysed using IBM SPSS Statistics 19.0 (IBM, Chicago, IL, USA) and MedCalc version 12.5.0 (Ostend, Belgium). The data were examined cross-sectionally and prospectively and explored as continuous variables and as binary categorical variables. Relationships between continuous variables were examined by Spearman correlation coefficients. χ^2^ test was used as a measure of association between categorical variables and odds ratios were used to examine the strength of associations. The cut-points used in this analysis to indicate cardiometabolic risk were ≥1.7 mmol L^−1^ for triglycerides, < 1.03 mmol L^−1^ for HDLc, ≥ 5.6 mmol L^−1^ for plasma glucose, ≥ 130 mmHg for systolic BP, and ≥85 mmHg for diastolic BP, as recommended by the International Diabetes Federation for children and adolescents (10 to 16 years) 24. The cut-points for LDLc and insulin were ≥2.79 mmol L^−1^ and ≥16.95 IU L^−1^, respectively, which is ≥90th centile for the cohort 2. Cardiometabolic risk factor co-occurrence was defined as having three or more cardiometabolic risk factors using the binary outcome thresholds listed above. Participants were classified as overweight or obese based on sex- and age- specific International Obesity Task Force (IOTF) BMI criteria 25 and ≥0.5 for WHtR 7. Receiver operator characteristic (ROC) curves were used to identify the optimal WHtR cut-points, sensitivity and specificity.

## Results

The anthropometric characteristics of participants are shown in Table 1. The prevalence of overweight or obese was lower in childhood (13.8%; *n* = 375) than in adolescence (17.2%; *n* = 490). The correlation between BMI z scores in childhood and adolescence was r = 0.72, *P* < 0.001, and 63.6% of overweight and obese children remained overweight or obese at 15 years. The proportion of participants who had a high WHtR (≥0.5) was also lower in childhood (6.8%; *n* = 185) compared with adolescence (17.2%; *n* = 492). The correlation between WHtR in childhood and adolescence was r = 0.57, *P* < 0.001 and 69.2% of children with a high WHtR had a high WHtR as adolescents.

**Table 1:** Anthropometric characteristics in childhood and adolescence. Results are expressed as median and interquartile range unless otherwise indicated

Childhood	Male		Female		Total	
	*n*		*n*		*n*	
Age (months)	1317	89 [89, 90]	1393	89 [89, 90]	2710	89 [89, 90]
Height (cm)	1315	127.0 [123.1, 130.9]	1393	125.7 [122.0, 130.1]	2708	126.5 [122.5, 130.6]
Height z score	1315	0.38 [−0.36, 1.01]	1393	0.23 [−0.49, 0.90]	2708	0.28 [−0.42, 0.95]
Weight (kg)	1317	25.4 [23.2, 28.6]	1393	25.2 [22.8, 28.8]	2710	25.4 [23.0, 28.6]
Weight z score	1317	0.26 [−0.35, 0.94]	1393	0.15 [−0.45, 0.86]	2710	0.20 [−0.41, 0.90]
BMI	1315	15.77 [14.92, 16.88]	1393	15.94 [14.90, 17.40]	2708	15.87 [14.91, 17.14]
BMI z score	1315	0.08 [−0.54, 0.71]	1393	0.04 [−0.58, 0.76]	2708	0.06 [−0.57, 0.74]
Overweight and obese, *n* (%)	1315	149 (11.3)	1393	226 (16.2)	2708	375 (13.8)
WHtR*	1314	0.44 [0.43, 0.46]	1392	0.44 [0.42, 0.46]	2706	0.44 [0.42, 0.46]
WHtR* ≥0.5, *n* (%)	1314	82 (6.2)	1392	103 (7.4)	2706	185 (6.8)

**Adolescence**	**Male**		**Female**		**Total**	
	***n***		***n***		***n***	
Age (months)	1376	184 [183, 186]	1482	184 [183, 187]	2858	184 [183, 187]
Height (cm)	1376	174.9 [170.0, 180.0]	1482	164.6 [161, 168.7]	2858	169.0 [163.5, 175.5]
Height z score	1376	0.47 [−0.16, 1.10]	1482	0.29 [−0.32, 0.96]	2858	0.37 [−0.22, 1.03]
Weight (kg)	1375	62.6 [56.8, 69.7]	1477	57.4 [52.2, 63.8]	2852	60.1 [53.9, 66.8]
Weight z score	1375	0.46 [−0.10, 1.03]	1477	0.36 [−0.29, 1.04]	2852	0.42 [−0.19, 1.03]
BMI	1375	20.38 [8.90, 22.24]	1477	21.10 [19.41, 23.23]	2852	20.71 [19.12, 22.80]
BMI z score	1375	0.32 [−0.30, 0.98]	1477	0.34 [−0.32, 1.01]	2852	0.33 [−0.31, 0.99]
Overweight or obese, *n* (%)		223 (16.2)		267 (18.1)		490 (17.2)
WHtR*	1376	0.43 [0.41, 0.46]	1482	0.46 [0.43, 0.49]	2858	0.44 [0.41, 0.48]
WHtR* ≥0.5, *n* (%)		152 (11.0)		340 (22.9)		492 (17.2)

* Waist-to-height ratio.

BMI, body mass index; WHtR, waist-to-height ratio.

BMI z scores and WHtR were highly correlated in both childhood and adolescence: *r* = 0.80 and *r* = 0.78 (both *P* < 0.001), respectively. The majority (91.9%) of children with a high WHtR were also overweight or obese. Conversely, only 45.1% of overweight or obese children had a high WHtR. In adolescence 72.0% of adolescents with a high WHtR were overweight or obese, and 71.8% of overweight or obese adolescents had a high WHtR.

The prevalence of elevated cardiometabolic risk factors in the adolescents varied from 2.2% (*n* = 62) for high diastolic BP, to 29.0% (*n* = 806) with high systolic BP, Table 2. In general, elevated cardiometabolic risk factors were more common in males than females, with approximately twice as many males having low HDLc, high glucose levels or high systolic BP compared to females. Co-occurrence of three or more cardiometabolic risk factors was more common in males than females. Correlations were stronger between cardiometabolic risk factors and anthropometric outcomes measured in adolescence compared to those measured in childhood, Table 3.

**Table 2:** Biochemistry and blood pressure in adolescence. Data are expressed as median [interquartile range] and the number (%) above cut-points, or below cut-point for HDLc

	Male		Female		Total		*P*†
	*n*		*n*		*n*		
Triglycerides (mmol L^−1^)	1376	0.73 [0.57, 0.96]	1482	0.77 [0.62, 0.99]	2858	0.75 [0.59, 0.98]	
≥1.7 mmol L^−1^		43 (3.1)		43 (2.9)		86 (3.0)	0.727
LDLc (mmol L^−1^)	1376	1.94 [1.62, 2.30]	1482	2.12 [1.79, 2.52]	2858	2.03 [1.70, 2.41]	
≥2.79 mmol L^−1^		104 (7.6)		221 (14.9)		325 (11.4)	<0.001
HDLc (mmol L^−1^)	1376	1.20 [1.01, 1.38]	1482	1.33 [1.16, 1.54]	2858	1.27 [1.09, 1.47]	
<1.03 mmol L^−1^		347 (25.2)		178 (12.0)		525 (18.4)	<0.001
Glucose (mmol L^−1^)	1376	5.3 [5.0, 5.5]	1482	5.1 [4.9, 5.3]	2858	5.2 [5.0, 5.4]	
≥5.6 mmol L^−1^		313 (22.7)		159 (10.7)		472 (16.5)	0.004
Insulin (IU L^−1^)	1374	8.17 [6.00, 11.16]	1480	9.65 [7.25, 12.76]	2854	8.97 [6.63, 12.05]	
≥16.95 IU L^−1^		102 (7.4)		155 (10.5)		257 (9.0)	<0.001
Systolic BP (mmHg)	1335	126.5 [119.0, 133.5]	1444	120.5 [113.0, 127.5]	2779	123.5 [115.5, 131.0]	
≥130 mmHg		517 (38.7)		289 (20.0)		806 (29.0)	<0.001
Diastolic BP (mmHg)	1335	66.5 [60.5, 73.5]	1444	66.0 [60.5, 71.0]	2779	66.0 [60.5, 72.0]	
≥85 mmHg		40 (3.0)		22 (1.5)		62 (2.2)	0.009
Risk co-occurrence*		104 (7.6)		64 (4.3)		168 (5.9)	<0.001

* Risk co-occurrence is the presence of ≥3 of the following: triglycerides ≥1.7 mmol L^−1^, LDLc ≥2.79 mmol L^−1^, HDLc <1.03 mmol L^−1^, plasma glucose ≥5.6 mmol L^−1^, insulin ≥16.95 IU L^−1^, systolic blood pressure ≥130 mmHg, or diastolic blood pressure ≥85 mmHg.

† Pearson χ^2^.

BP, blood pressure; HDLc, high density lipoprotein cholesterol; LDLc, low density lipoprotein cholesterol.

**Table 3:** Spearman correlations (*P* value) between BMI z score and waist-to-height ratio measured in childhood and adolescence and cardiometabolic risk factors measured in adolescence

Cardiometabolic risk factors	Childhood				Adolescence			
	BMI z score		WHtR		BMI z score		WHtR	
	Male	Female	Male	Female	Male	Female	Male	Female
Triglycerides (mmol L^−1^)	0.083 (0.003)	0.052 (0.055)	0.077 (0.005)	0.079 (0.003)	0.270 (<0.001)	0.145 (<0.001)	0.321 (<0.001)	0.185 (<0.001)
LDLc (mmol L^−1^)	0.073 (0.008)	0.102 (<0.001)	0.096 (<0.001)	0.120 (<0.001)	0.119 (<0.001)	0.158 (<0.001)	0.148 (<0.001)	0.161 (<0.001)
HDLc (mmol L^−1^)	−0.137 (<0.001)	−0.101 (<0.001)	−0.143 (<0.001)	−0.096 (<0.001)	−0.271 (<0.001)	−0.242 (<0.001)	−0.211 (<0.001)	−0.260 (<0.001)
Glucose (mmol l^−1^)	0.001 (0.964)	−0.015 (0.570)	0.012 (0.661)	−0.014 (0.597)	0.077 (0.004)	−0.004 (0.888)	0.092 (0.001)	−0.116 (0.542)
Insulin (IU L^−1^)	0.119 (<0.001)	0.111 (<0.001)	0.099 (<0.001)	0.105 (<0.001)	0.284 (<0.001)	0.192 (<0.001)	0.352 (<0.001)	0.189 (<0.001)
Systolic BP (mmHg)	0.129 (<0.001)	0.139 (<0.001)	0.060 (0.033)	0.103 (<0.001)	0.228 (<0.001)	0.228 (<0.001)	0.148 (<0.001)	0.183 (<0.001)
Diastolic BP (mmHg)	0.025 (0.368)	0.003 (0.912)	−0.044 (0.119)	−0.063 (0.021)	0.031 (0.257)	0.0.21 (0.417)	−0.022 (0.429)	0.015 (0.576)

BMI, body mass index; BP, blood pressure; HDLc, high density lipoprotein cholesterol; LDLc, low density lipoprotein cholesterol; WHtR, waist-to-height ratio.

**Figure 1:**
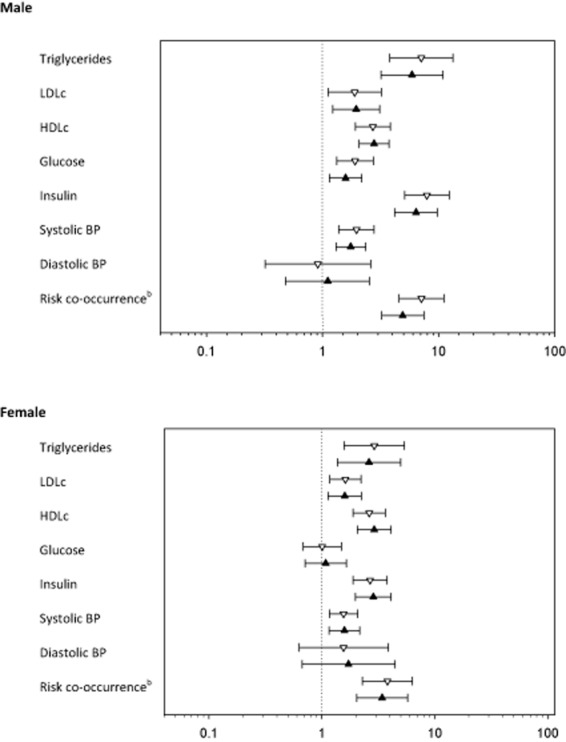
Odds ratio (95%CI) for risk factors associated with overweight and obesity defined by BMI (*symbol: black triangle*) and WHtR of ≥0.5 (*symbol: white triangle*) in adolescents (cross-sectional analysis).Risk factors were defined as follows: triglycerides ≥1.7 mmol/L, LDLc ≥ 2.79 mmol/L, HDLc < 1.03 mmol/L, plasma glucose ≥5.6 mmol/L, insulin ≥16.95 IU/L, systolic blood pressure ≥130 mmHg, or diastolic blood pressure ≥85 mmHg. ^b^Risk co-occurrence was defined as ≥3 of the above.

The proportions of overweight and obese adolescents, or adolescents with a WHtR ≥0.5, that had elevated LDLc, reduced HDLc, elevated glucose, elevated insulin and/or high BP are shown in Table 4. The odds ratios between measures of adiposity and risk factors are shown in Fig. 1. Adolescent males with WHtR ≥0.5 had an increased odds of elevated triglycerides, LDLc, glucose, insulin and systolic BP and low HDLc compared to males with a WHtR <0.5. Furthermore, males with WHtR ≥0.5 had an increased odds ratio of co-occurrence of cardiometabolic risk factors compared to males with a WHtR <0.5 (odds ratio = 6.8 [95% confidence interval {CI} 4.4 to 10.6]). Overweight and obese males had correspondingly increased odds of a similar magnitude, compared with males who were not overweight or obese. Females with a high WHtR had increased odds ratio for elevated triglycerides, LDLc, insulin and systolic BP and low HDLc, and were approximately four times more likely (3.8 [2.3 to 6.3]) to have co-occurrence of cardiometabolic risk factors than females with a WHtR <0.5. Overweight and obese females had correspondingly increased odds of the same cardiometabolic risk factors and co-occurrence of these, (Fig. 1).

**Table 4:** The proportion of adolescents with cardiometabolic risk factors, stratified by sex and anthropometric status (cross sectional analysis)

Outcome and exposure	Male			Female		
	% with increased adiposity* and risk factor/s	% without increased adiposity* and with risk factor/s	*P*^b^	% with increased adiposity* and risk factor/s	% without increased adiposity* and with risk factor/s	*P*†
Triglycerides (≥1.7 mmol L^−1^)						
BMI	9.9	1.8	<0.001	5.6	2.2	0.003
WHtR	12.5	2.0	<0.001	2.9	2.0	0.001
LDLc (≥2.79 mmol L^−1^)						
BMI	12.1	6.6	0.004	20.2	13.7	0.007
WHtR	12.5	6.9	0.013	20.3	13.3	0.002
HDLc (<1.03 mmol L^−1^)						
BMI	43.5	21.7	<0.001	23.2	9.4	<0.001
WHtR	44.1	22.9	<0.001	21.2	9.3	<0.001
Glucose (≥5.6 mmol L^−1^)						
BMI	30.0	21.4	0.005	11.2	10.5	0.72
WHtR	34.2	21.3	<0.001	10.9	10.7	0.97
Insulin (≥16.95 IU L^−1^)						
BMI	22.9	4.4	<0.001	8.2	7.4	<0.001
WHtR	28.3	4.8	<0.001	18.9	8.0	<0.001
Systolic BP (≥130 mmHg)						
BMI	50.2	36.6	<0.001	26.5	18.6	0.003
WHtR	52.7	37.0	<0.001	25.6	18.3	0.004
Diastolic BP (≥85 mmHg)						
BMI	3.3	2.9	0.81	2.3	1.4	0.26
WHtR	2.7	3.0	0.85	2.1	1.3	0.31
Risk co-occurrence‡						
BMI	20.6	5.0	<0.001	9.7	3.1	<0.001
WHtR	27.0	5.1	<0.001	9.7	2.7	<0.001

* BMI was categorized as overweight/obese or not overweight/obese and WHtR was categorized as <0.5 or ≥0.5.

† Pearson χ^2^.

‡ Risk co-occurrence is the presence of ≥3 of the following: triglycerides ≥1.7 mmol L^−1^, LDLc ≥2.79 mmol L^−1^, HDLc <1.03 mmol L^−1^, plasma glucose ≥5.6 mmol L^−1^, insulin ≥16.95 IU L^−1^, systolic blood pressure ≥130 mmHg, or diastolic blood pressure ≥85 mmHg.

BMI, body mass index; BP, blood pressure; HDLc, high density lipoprotein cholesterol; LDLc, low density lipoprotein cholesterol; WHtR, waist-to-height ratio.

The specificity and sensitivity of a WHtR cut-point of 0.5 in male adolescents for identifying cardiometabolic risk factor co-occurrence was 90.0 (95% CI 88.2 to 91.6) and 41.3 (31.8 to 51.4), respectively. For female adolescents the specificity and sensitivity of a WHtR cut-point of 0.5 was 76.2 (73.7 to 78.2) and 53.1 (40.2 to 65.7). Using ROC analysis the optimal WHtR cut point for identifying co-occurrence of risk factors was 0.47 (specificity 83.5 [81.3 to 85.5]; sensitivity 51.9 [41.9 to 61.8]) for male adolescents and 0.48 (specificity 65.8 [63.3 to 68.3]; sensitivity 67.2 [54.3 to 78.4] for female adolescents.

**Figure 2:**
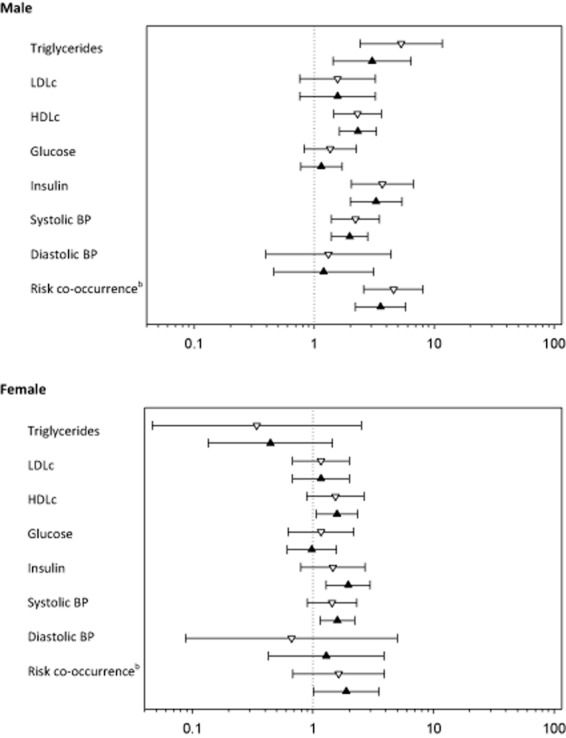
Odds ratio (95% confidence interval) for risk factors associated with overweight and obesity defined by body mass index (▴) and waist-to-height ratio of ≥0.5 (▽) in childhood (prospective analysis). Risk factors were defined as follows: triglycerides ≥1.7 mmol/L, low density lipoprotein cholesterol (LDLc) ≥2.79 mmol/L, high density lipoprotein cholesterol (HDLc) <1.03 mmol/L, plasma glucose ≥5.6 mmol/L, insulin ≥16.95 IU/L, systolic blood pressure (BP). ^b^Risk co-occurrence was defined as ≥3 of the above.

The proportions of overweight and obese children or those with a WHtR ≥0.5 who had elevated LDLc, reduced HDLc, elevated glucose, elevated insulin and/or high BP during adolescence are shown in Table 5. Like the cross-sectional analysis, male children who had a WHtR ≥ 0.05 or who were overweight or obese had increased odds of cardiometabolic risk factors in adolescence, Fig. 2. However, the CIs around the odds ratio tended to be wider. Male children with WHtR ≥0.5 had approximately five times higher probability (4.6 [2.6 to 8.1]) of co-occurrence of risk factors during adolescence than male children with a WHtR <0.5. Overweight and obese male children had approximately four times higher probability (3.6 [2.2 to 5.8]) of co-occurrence of risk factors during adolescence. Similar associations between both anthropometric measures and cardiometabolic risk factors were also observed in female children, although the odds ratios were generally small, and the lower limits of the CIs below one, Fig. 2.

**Table 5:** The proportion of adolescents with cardiometabolic risk factors, stratified by sex and anthropometric status in childhood (prospective analysis)

Outcome and exposure	Male			Female		
	% with increased adiposity* and risk factor/s	% without increased adiposity* and with risk factor/s	*P*†	% with increased adiposity* and risk factor/s	% without increased adiposity* and with risk factor/s	*P*†
Triglycerides (≥1.7 mmol L^−1^)						
BMI	6.7	2.3	0.002	1.3	2.9	0.17
WHtR	11.0	2.3	<0.001	1.0	2.8	0.27
LDLc (≥2.79 mmol L^−1^)						
BMI	10.0	7.2	0.22	17.0	14.2	0.58
WHtR	11.0	7.3	0.22	16.5	14.5	0.58
HDLc (<1.03 mmol L^−1^)						
BMI	40.7	23.6	<0.001	16.6	10.8	0.02
WHtR	42.7	24.4	<0.001	16.5	11.4	0.12
Glucose (≥5.6 mmol L^−1^)						
BMI	24.7	22.5	0.50	10.4	9.6	0.92
WHtR	28.0	22.3	0.23	11.7	10.2	0.63
Insulin (≥16.95 IU L^−1^)						
BMI	16.1	5.8	<0.001	14.5	8.3	0.001
WHtR	19.8	6.2	<0.001	13.7	9.0	0.22
Systolic BP (≥130 mmHg)						
BMI	53.1	36.9	<0.001	27.5	18.9	0.006
WHtR	57.0	37.5	0.001	25.3	19.9	0.13
Diastolic BP (≥85 mmHg)						
BMI	3.4	2.9	0.71	1.8	1.4	0.65
WHtR	3.8	2.9	0.66	1.0	1.5	0.69
Risk co-occurrence‡						
BMI	18.1	6.1	<0.001	6.3	3.4	0.04
WHtR	24.4	6.4	<0.001	6.1	3.7	0.27

* BMI was categorized as overweight/obese or not overweight/obese and WHtR was categorized as <0.5 or ≥0.5.

† χ^2^ (Pearson).

‡ Risk co-occurrence is the presence of ≥3 of the following: triglycerides ≥1.7 mmol L^−1^, LDLc ≥2.79 mmol L^−1^, HDLc <1.03 mmol L^−1^, plasma glucose ≥5.6 mmol L^−1^, insulin ≥16.95 IU L^−1^, systolic blood pressure ≥130 mmHg, or diastolic blood pressure ≥85 mmHg.

BMI, body mass index; BP, blood pressure; HDLc, high density lipoprotein cholesterol; LDLc, low density lipoprotein cholesterol; WHtR, waist-to-height ratio.

The specificity and sensitivity of a WHtR cut-point of 0.5 in male children for identifying cardiometabolic risk factor co-occurrence in adolescents was 94.7 (93.3 to 95.9) and 21.1 (13.4 to 30.6), respectively. For female children the specificity and sensitivity of a WHtR cut-point of 0.5 was 91.4 (89.8 to 92.9) and 17.0 (8.1 to 29.8). Using ROC analysis the optimal WHtR cut point for identifying co-occurrence of risk factors in male children was the same as it was in male adolescents 0.47 (specificity 83.3 [81.0 to 85.3]; sensitivity 37.9 [28.1 to 48.4]), but lower in female children compared to female adolescents 0.44 (specificity 54.4 [51.7 to 57.1]; sensitivity 71.7 [57.7 to 83.2]).

## Discussion

To our knowledge this is the first study to demonstrate a prospective association between having a high WHtR in childhood and cardiometabolic risk co-occurrence in adolescent boys. The results demonstrate that the of risk for having a WHtR ≥0.5 was comparable to being overweight or obese as defined by sex- and age- specific IOTF BMI criteria. For example, children with a WHtR ≥0.5, had two to five times higher odds of cardiometabolic risk co-occurrence compared to children who had a WHtR <0.5 and children who were overweight or obese had two to four times higher odds compared to normal weight children. However, there was a sex difference and the association between anthropometric measures in childhood and cardiometabolic risk co-occurrence in adolescence was significantly stronger in boys than girls. In the clinical environment, using a WHtR cut-point of 0.5 has several advantages over defining a child as overweight or obese by BMI; it is a simpler index to calculate, easily understood by adolescents and families and does not require sex- and age- specific centiles.

The findings of this study are broadly consistent with another prospective study that examined correlations between BMI and WHtR in Australian children and adult cardiometabolic risk factors 20. While direct comparisons between studies is difficult, it is interesting to note that the correlations between WHtR and BMI measured at 7 to 11 years with triglycerides (rho = 0.07 and 0.05 for WHtR and BMI, respectively) and insulin (rho = 0.11 for both WHtR and BMI) measured in adulthood were of a similar magnitude to what we report between WHtR and BMI measured at 7 to 9 years and triglycerides and insulin measured in adolescence.

The association between WHtR and cardiometabolic risk, both measured in adolescence, support a number of other cross-sectional studies 26,27, which have been recently reviewed 7. In general, all studies showed good agreement in the magnitude and outcome of their analysis between WHtR and BMI and outcomes of cardiometabolic risk. Consistent with our study, both WHtR and BMI had a stronger association with triglycerides and HDLc, than LDLc. The association between anthropometric measures and BP tended to be stronger for systolic BP compared to diastolic BP and both WHtR and BMI were associated with fasting insulin and a ‘cardiometabolic risk score’, albeit defined differently among the different studies.

There is no consensus on the cross-sectional association of WHtR and BMI and fasting glucose 3,15,17. In part, this may be explained by the differing age of the cohorts (range from 4 to 17 years) and methods of analysing the data. Findings from our study suggest that adolescent males with a high WHtR or who are overweight or obese have an increased risk of an elevated fasting glucose. However, it is of note that in our study a high proportion of adolescents (23% of males; 11% of females) had fasting blood glucose levels ≥5.6mmol L^−1^. In the absence of evidence to indicate that this population has disturbances in glucose metabolism, these findings could reflect the diurnal variation in blood glucose 28, since some adolescents had blood taken in the afternoon after a 6 h fast and/or inadequate fasting. The results pertaining to fasting glucose in this study need to be interpreted with caution.

More children were classified as overweight or obese using BMI criteria compared to those classified as having a high WHtR. Children with a WHtR ≥0.5 were more severely obese compared to children were defined as overweight or obese using BMI criteria, mean ± standard deviation BMI z-score was 2.05 ± 0.9 and 1.82 ± 0.9, respectively. The most appropriate cut-point for WHtR to identify children and adolescents at cardiometabolic risk is not clear. In the present study we used ≥0.5 which has demonstrated utility in adults 7 and children 10 but other cut-points have been suggested 8. Our results indicated that a cut-point ≥0.5 was highly specific in identifying cardiometabolic risk co-occurrence in male children and adolescents as well as female children (90 to 95%), but had poor sensitivity (17 to 53%); the risk of being wrongly labelled as having co-occurrence of cardiometabolic risk factors was small, but many of those who were at risk were not identified. Statistically optimum cut-points were identified as 0.47 for male children and male adolescents and 0.44 and 0.48 for female children and female adolescents, respectively. However, by increasing the sensitivity, the specificity decreased particularly for female children (from 91 to 54%); using lower cut-points more children and adolescents would be incorrectly labelled as being as having co-occurrence of cardiometabolic risk factors. Given the stigma of being incorrectly labelled and the limited resources for managing children and adolescents at cardiometabolic risk it could be prudent to use a higher but more specific cut-point.

There are a number of limitations to the study including the number of participants which had complete data. Initially over 14 000 pregnant women were recruited, with over 5500 participants attending the 15 years clinic, yet only 2710 met our inclusion criteria for our prospective analysis and 2858 for our cross-sectional analysis. The implications of attrition are not clear and may affect the generalizability of the findings. Nevertheless, this is the largest study in children and adolescents addressing the association between WHtR and cardiometabolic risk factors, as well as the first in the UK.

Another potential limitation relates to measuring waist circumference and applies to both the location of the measurement and sensitivity around measuring waist circumference in heavier children. There is no universally accepted method of measuring waist circumference. In this study the waist circumference was measured at the mid-point between the lowest rib and the iliac crest, which has been demonstrated to be reproducible and highly correlated with total body and trunk adiposity 29. It is recognized that removal of clothing and placement of the measuring tape may be awkward or embarrassing for the overweight or obese children and needs to be handled sensitively.

In conclusion, we found that BMI z scores and WHtR were highly correlated and that they have similar associations with cross-sectional and prospective cardiometabolic risk factors in adolescence. WHtR is a simple calculation that could be used to identify male children and adolescents for cardiometabolic risk.
